# Harnessing brain and cognitive reserve for the prevention of dementia

**Published:** 2009-01

**Authors:** Michael Valenzuela, Perminder S. Sachdev

**Affiliations:** School of Psychiatry, University of New South Wales, Sydney; Neuropsychiatric Institute, Prince of Wales Hospital, Randwick, Australia

**Keywords:** Brain reserve, cognitive exercise, cognitive reserve, cognitive training, dementia, prevention

## Abstract

The concepts of brain and cognitive reserve capture several elements of common wisdom – that we all differ in the neural resources we are endowed at birth, that experience and especially complex mental activities then modify how these neural resources are organized and cultivated, and that after any form of brain injury there is significant individual variation in the degree to which clinical deficits may manifest. Transforming these insights into a formal and refutable working definition, however, has been more challenging. Depending on the scale of analysis, brain and cognitive reserve have been defined from neurocentric, neuropsychological, computational, and behavioral perspectives. In our research, we have focused on the behavioral definition, whereby an individual’s lifetime exposure to complex mental activities is used for prediction of longitudinal cognitive and neurological change. This approach also benefits from a wealth of epidemiological studies linking heightened complex mental activity with reduced dementia risk. Research in the field of cognitive training is also beginning to indicate that incident cognitive decline can be attenuated, with recent clinical trials addressing the major challenges of transfer of gain and durability of effect. High quality randomized clinical trials are therefore the most urgent priority in this area so that the promise of brain and cognitive reserve can be harnessed for the purpose of the primary prevention of dementia.

## WHAT IS RESERVE?

While most neuroscientists and clinicians would agree that the brain has reserve capacity, the concept remains difficult to define. What exactly is it that researchers mean when invoking ‘reserve’? Part of the difficulty results from the fact that this question can be approached from many angles. These are briefly outlined below.

## NEUROCENTRIC PERSPECTIVE

Katzman *et al*.[1] first noted that cognitively normal individuals with elevated Alzheimer’s disease (AD) pathology had almost double the number of large pyramidal neurons throughout their neocortex in comparison to those who expressed clinical symptoms, suggesting that the lack of symptoms in the former was because of a ‘greater reserve’. The neurocentric version of brain reserve therefore implies an advantage based on increased neuronal numbers. This interpretation was also consistent with action of a linear threshold mechanism,[2] whereby either a larger static lesion, or the more protracted course of a progressive lesion, was required before an individual may cross below a hypothetical functional threshold.

Since neuronal counts are not possible in life, clinical studies have often relied on brain volume as a marker of such reserve capacity. Use of a gross measure such as whole brain volume, however, risks confusing a predictor variable (one’s estimate of reserve) with a known outcome variable (age-related cerebral atrophy). An alternative that has been explored in the context of dementia is intracranial volume (ICV) as a proxy for maximal brain volume, and even head circumference[3] and head width[4] as more basic measures. In general, these studies have failed to show a general association with dementia incidence across the full range of ICV, but rather an increased risk only in the low to very low ranges[5] or when in the presence of an additional risk factor such as ApoE4.[3]

An obvious problem for the neurocentric interpretation relates to the implication that brain reserve is a non-modifiable entity. Maximum ICV and head circumference are generally achieved by puberty[6] and reflect genetic variance in neuronal quantum as well as developmental, nutritional, and environmental factors in early life.[7] More importantly, since these measures do not generally change after the onset of adulthood, does this mean that underlying brain reserve can also not change? As will be reviewed below, the weight of evidence from the epidemiological, clinical, and experimental literature suggests the opposite. Dementia risk appears to be highly modifiable by experience well into late life. For these reasons, a neurocentric version of brain reserve is not tenable, although it is important to note that this reflects current technological limitations rather than any underlying conceptual flaw. Brain reserve, of whatever flavor, must at some level have a neurobiological correlate.

## COGNITIVE PERSPECTIVE

Rather than focus on the maximal quantity or volume of neurons, the *cognitive reserve* perspective is more interested in ‘how well we use what has been left behind’.[8,9] Two non-trivial distinctions have been made. First, we could replace “neuronal numbers” with “neuropsychological competence” or “intelligence”, and so apply a similar threshold mechanism whereby high *cognitive reserve* individuals simply perform better on cognitive tests to start with and therefore require a larger fall before reaching diagnostic threshold. Importantly, this account suggests no interaction with the underlying and progressive neurodegenerative process, and so predicts no differential rate in cognitive decline, only different starting points for the decline. It has therefore been termed a *passive* account[8] and been identified as a potential source of systematic error in longitudinal studies.[10] Clearly, in the context of ageing and dementia, any reserve definition must be able to independently predict differential rates of neuropsychological change, not just differences in incidence of impairment.

Alternatively, a more dynamic form of cognitive reserve suggests that individuals who have developed a range of deliberate cognitive strategies for solving complex problems, such as navigating around the neighborhood, and perform well on neuropsychological tests, are more likely to remain within normal limits for longer despite the parallel progression of underlying disease. This *active* account therefore suggests that two individuals may begin at the same neuropsychological starting point and suffer the same longitudinal burden of disease, but due to increased strategic mechanisms, one may perform better at follow-up testing, or suffer from less day-to-day symptoms. Whilst capturing part of the ecological nature of reserve, this definition is extraordinarily difficult to measure. Simply asking subjects about their use of deliberate strategies whilst performing memory tasks can, for example, produce more questions than answers.[11] On the other hand, if we assume that one’s capacity for deliberate cognitive flexibility is akin to greater problem solving or ability to set-shift, we again risk confounding a predictor with an outcome variable (i.e., age-related decline in executive functions).

## COMPUTATIONAL PERSPECTIVE

A more sophisticated approach to *reserve* revolves around the notions of *computational redundancy and flexibility*. High *cognitive reserve* individuals may not only have a wider repertoire of conscious and preconscious strategies, but also a greater number of potential neural pathways for execution of these cognitive processes, thus allowing maintenance of function despite neurological insult. A person with high reserve and high disease burden may therefore remain asymptomatic due to compensatory and resistant adaptations to functional brain networks; alternatively, a low reserve individual may express symptoms after only a trivial brain insult due to a lack of redundant neural pathways and an inflexible or intolerant functional network.[12,13]

Whilst this approach is attractive as it unifiesbrain and *cognitive reserve* with a specific mechanism, problems with operationalization again arise. Do we attempt to estimate neurocomputational factors such as network efficiency and redundancy via some type of functional neuroimaging ’stress test’? Whilst of high interest, a working definition of *brain reserve* along these lines is currently not practical.

## BEHAVIORAL PERSPECTIVE

The strategy adopted in our research has been a behavioral one and simply asks how mentally active and engaged a person is in comparison with the average? It therefore seeks a reliable definition at the level of day-to-day observable and verifiable facts related to *complex mental activity*. The type of information relevant to this version ofreserveincludes level and duration of formal education, the nature and complexity of occupational history, and the diversity, frequency and cognitive challenge of past and present leisure activities. This has been combined into a validated assessment tool, the *Lifetime of Experience Questionnaire* (LEQ),[14] which takes 15 min to complete and is now available online for research use (http://train.headstrongcognitive.com/leq.aspx). Higher LEQ scores independently predict not only attenuated cognitive decline over time, but also a reduced rate of hippocampal atrophy.[15] The main advantage from such a straightforward formulation is therefore the provision of an operational definition which is clinically relevant.

Importantly, this behavioral approach to measuring *brain* or *cognitive reserve* does not identify itself with a specific neurological quantum, computational property or cognitive process. It seems self evident that participation in complex mental activities will lead to changes in a number of interacting mechanisms at different temporal and spatial scales,[12] which together may alter an individual’s risk for dementia and cognitive dysfunction. In our opinion, there is no *one brain or cognitive reserve, but a number of reserves*.[16] Ourworking definitionis hence at the behavioral scale for essentially pragmatic reasons because it is arguably more amenable to precise characterization and hence general utility.

## LINKS BETWEEN MENTAL EXERCISE AND DEMENTIA RISK

Several large-scale epidemiological studies have linked participation in complex mental activity to reduced dementia risk. A systematic review of 22 longitudinal cohort studies found that individuals with higher levels of education, occupation or engagement in complex cognitive activities were at 46% lower risk for incident dementia than those with low levels.[17]

When restricting analysis to those cohort studies of older persons that have focused on cognitive complexity in their current lifestyle, protective effects remain significant even after controlling for earlier life exposures such as education, occupation, baseline cognition, and cardiovascular risk factors.[18–22] Moreover, evidence for a dose dependent relationship is evident from studies that show corresponding decrements in dementia risk as a function of increasing participation in cognitive lifestyle activities.[23] For example, Verghese *et al*. found that compared to those older individuals with the lowest level of participation in cognitive lifestyle activities, those with moderate activity levels had 50% lower risk for dementia over 5 years follow-up, and those with the highest activity levels had 67% lower risk for incident dementia.[24]

There is now highly consistent epidemiological support for an association between complex mental activity and dementia risk, but the *nature of the relationship* remains contentious for two main reasons. First, the biological mechanisms which may mediate a nexus between mental activity and dementia are not clear. Some potential mediating mechanisms are briefly reviewed below. Second, and more importantly, there are questions about the *direction of causality*: does preceding mental activity reduce or delay expression of future dementia or is preclinical dementia causing a reduction in participation in activities prior to formal diagnosis? To disentangle this complex ‘chicken or egg’ problem, data from clinical trials using *cognitive training* interventions are required.

## CLINICAL TRIALS OF COGNITIVE TRAINING

### Definition of cognitive training

Cognitive training is any intervention aimed at improving, maintaining, or restoring mental function through the repeated and structured practice of tasks which pose an inherent problem or mental challenge. Importantly, this definition does not include training in strategies to compensate for deficits, traditionally a rehabilitative or remedial approach.[25]

## RANDOMIZED CONTROLLED TRIALS IN LATE LIFE

Repetitive cognitive training undoubtedly improves performance *on the trained task* – there is indeed more than 20 years of cognitive psychology research on this topic.[26] To determine whether cognitive training could potentially help reduce or delay the incidence of dementia, two major issues need consideration:

1. Generalization or transfer of effect
  Does the cognitive training intervention only lead to improvement in the trained task, or does it also transfer to non-trained tasks? We propose a hierarchy of generalization of increasing clinical relevance:
  - Transfer to non-trained tasks in same cognitive domain
  - Transfer to non-trained tasks in other cognitive domains
  - Transfer to global measures of general cognitive ability (e.g., Alzheimer’s disease assessment scale - cognitive, tests for general intellectual ability, etc.)
  - Transfer to measures of general function (e.g., instrumental activities of daily living, quality of life, mood, etc.)
2. Persistence or durability of effect
  Does the effect of cognitive training intervention last beyond the immediate post-training period, or is continual cognitive training required? Longitudinal follow-up of cognitive training efficacy is required to answer this question.

We have recently published a systematic review of randomized controlled trials (RCT) of cognitive training in healthy older individuals in which longitudinal follow-up was a critical design feature.[27] The results of seven identified trials suggest that a discrete program of cognitive exercise in the order of 2-3 months may have long-lasting and persistent protective effects on cognition over a number of years. The overall weighted mean difference was strong in magnitude, estimated at 1.07 (CI: 0.32–1.83); the overall non-weighted average Cohen’s d effect size was 0.5.

The ACTIVE study is the largest trial in the area[28] and examined the effects of 10 sessions of cognitive training on 2832 healthy older individuals. Participants completed three different intervention groups: memory training, reasoning training, and processing speed training. Two years later, each intervention improved cognitive ability in the targeted area. Importantly, at this stage there was no evidence of transfer of gain to other domains, nor any effect on instrumental activities of daily living. Follow up at five-years, however, found that reasoning training specifically protected against functional decline compared to any of the other interventions or the control wait-and-see condition.[29] This is therefore the first large clinical trial to demonstrate transfer of effect since cognitive training produced enduring benefits on a *general functional outcome* that is highly relevant to dementia onset.

There is also a high degree of community and commercial interest in computer-based cognitive training. One group has conducted a RCT with one such product in a report that included co-authors from the parent company.[30] The attraction of computerized cognitive training is that training can be standardized and allows a gradient in task difficulty to be incorporated as individuals’ skill levels progress. Neuropsychological tests immediately after the end of the training period found verbal memory performance improved by up to 25% of a standard deviation, and testing 3 months later showed that short-term memory performance remained enhanced.

The combination of both cognitive and physical exercise is also of great interest. This has yet to be tested in a rigorous RCT. The Sim-A study investigated the effects of cognitive, physical, and combined training in healthy older individuals over a five year period.[31] Thirty paper-and-pencil cognitive training sessions produced a significant effect over both the 12 month and 5–year follow–up periods. Moreover, this effect seemed to transfer to a measure of general cognition. The group that did both cognitive and physical training experienced a larger effect size than those who completed just one type of training, suggesting a potential for synergy between these modalities. Other smaller studies with samples of less than 100 individuals have found positive trends but have lacked power.[32,33]

The overall effect size and consistency across longitudinal trials of cognitive training is therefore promising, yet many questions remain. There has been a wide variety of primary outcome measures, for example, across the trials, and details of the applied cognitive tasks also varied. Quality of trial design and reporting has in general been low.

It is however encouraging that those studies with longer term follow–up showed no evidence of less potent effects. A durable long-term effect from cognitive training may therefore be realistic. Finally, two of the more recent clinical studies have shown that their training protocols generalize to domains beyond the narrow focus of the trained tasks.[29,31] Well designed cognitive training interventions may thereby have the potential to contribute to primary prevention of dementia as part of a holistic risk reduction strategy.

## HOW COULD MENTAL EXERCISE PREVENT DEMENTIA?

Complex mental activity clearly alters the structure and function of the brain on a number of levels. We have previously reviewed how the spatial and temporal nature of these changes may contribute to defense against dementia,[12] with information gained mainly from decades of work studying the effects of enrichment on the rodent brain[34] and from human brain imaging. Here, we present a highly condensed summary.

### 

#### Molecular mechanisms

Short periods of increased activity alter long-term potentiation and depression,[35] possibly by upregulation of AMPA receptors.[36] Neurotrophic hormones are also increased, particularly BDNF[37,38] and NGF.[39] Strikingly, transgenic Alzheimer model mice show dramatic decrements in pathology[40–42] and improvements in behavior[43] after several weeks of enrichment compared to control littermates. The time frame for detection of biological changes secondary to enrichment is minimal, given that microarray analysis has shown dozens of gene expression changes following as little as three hours of exposure.[44]

#### Cellular mechanisms

Synaptogenesis is arguably the most robust cellular change, in the order of 150-300%,[45] and potentially the most clinically relevant, as decline in synaptic density is strongly correlated with cognitive status in dementia.[46] Experience-dependent neurogenesis[47] and angiogenesis[48] also occur, which in combination may explain why enrichment seems to lead to increased gross brain volume,[7] as well as increased regional volumetric changes in humans.[49,50]

#### Cortical network mechanisms

Repeated cognitive exercise leads to increased metabolic efficiency across whole brain networks,[51] whilst selectively and temporarily increasing haemodynamic responsivity in brain areas engaged by the cognitive tasks.[52,53] There is also evidence for an enhanced ability to adapt and compensate against progressive disease in the medial temporal lobe, particularly through functional reorganization in the prefrontal cortex.[54–57]

### Recommendations and challenges for the future

A confluence of research streams has emerged in the last 10 years as the basis for intense medical, community and commercial interest in trying to harness the power of brain and cognitive reserve for the prevention of age–related cognitive dysfunction. Perhaps the greatest influence has been a wider sociological trend away from pharmacological agents and towards behavioral and lifestyle modification and positivistic health attitudes. Yet this enthusiasm should not obscure our demand for rigorous scientific evidence when attempting to translate preclinical findings to individual and community–wide interventions. The greatest challenges for the area are therefore in the domain of clinical trials. No trial has, for example, definitively shown that cognitive training reduces the incidence of dementia, as opposed to the rate of cognitive decline. Higher caliber RCTs are required, with close attention to design of control groups, longitudinal evaluation, choice of cognitive training protocol and outcome measures, and recruitment of relevant samples.

This information will then need to inform wider community programs, and many more questions arise. For example, is starting a new cognitively demanding hobby as good as many hours of computer–based cognitive training? If so, are all activities equally effective or only some? How often and what intensity of engagement is required? Is group participation better than individual practice at home? Generic issues will also arise such as scalability, accessibility, economy, and accountability.

In the meantime, the general public needs to be well informed about the links between complex mental activity and reduced dementia risk. Given the negligible potential for harm, it is sensible to encourage all individuals to increase their levels of complex, enjoyable, and engaging cognitive activity for optimal brain health, particularly after retirement.
